# Prevalence and correlates of antinuclear antibodies in a Japanese community sample: a cross-sectional study

**DOI:** 10.1016/j.curtheres.2026.100845

**Published:** 2026-09-16

**Authors:** Tamanna Tabassum, Yoshiki Kuroda, Tatsuyoshi Ikenoue, Takuji Hinoura

**Affiliations:** Department of Public Health, Faculty of Medicine, University of Miyazaki, Miyazaki, Japan

**Keywords:** Antinuclear antibodies, Prevalence, Healthy individuals, Sex factors, Indirect immunofluorescence, Japan

## Abstract

**Background:**

Antinuclear antibodies (ANA) are among the most commonly used tests in autoimmune disease but are frequently positive in healthy individuals, with uncertain significance. Reported prevalence ranges from under 6% to over 45% across populations, and its determinants in healthy individuals are poorly defined.

**Objective:**

We examined ANA prevalence and its demographic and lifestyle correlates in a community sample in Japan.

**Methods:**

Serum samples were obtained from 1,017 community residents in a single prefecture in Japan. Age and sex were recorded for all participants; smoking status and allergic history were available for a subset (n = 467). ANA were measured by indirect immunofluorescence on HEp-2 cells; a 1:40 titer defined positivity. Associations were assessed using chi-square tests, logistic regression with linear and quadratic age terms, and multivariable logistic regression adjusting sequentially for age, sex, smoking, and allergic history.

**Results:**

Among 1,017 participants (542 women and 475 men), overall ANA positivity was 18.3% (186/1,017). Positivity was significantly higher in women than in men (21.8% vs. 14.3%) and showed a U-shaped age pattern in women. ANA positivity was not associated with allergic history. Exploratory subgroup analysis showed higher positivity in current or former smokers than never-smokers among men. After adjustment for age, female sex was independently associated with ANA positivity (OR 1.72, 95% CI 1.24–2.40). In the lifestyle subset, female sex was the only independently associated factor after adjustment for age, smoking status, and allergic history.

**Conclusions:**

These data provide a population-specific baseline for ANA positivity in a Japanese community sample and confirm that prevalence is higher in women and varies with age. Because ANA is frequently measured and may precede autoimmune disease, longitudinal studies are needed to clarify whether incidental positivity in healthy individuals carries prognostic significance.

## Introduction

Antinuclear antibodies (ANA) are established biomarkers that aid in diagnosing autoimmune diseases and predicting their prognosis. They are widely used to evaluate connective tissue diseases such as systemic lupus erythematosus (SLE), systemic sclerosis, and Sjögren’s syndrome,^1^^,^^2^ as well as various other conditions.^3^ Several laboratory methods are available for ANA detection, and indirect immunofluorescence (IIF) using human epithelial type 2 (HEp-2) cells is considered the gold standard. IIF is commonly used in clinical settings because of its simplicity, relatively low cost, and high sensitivity and specificity. This method provides clinically meaningful information on specific autoantibody profiles.^4^^,^^5^

However, ANA positivity is not limited to autoimmune diseases; it has also been documented in malignancies^6^ and cardiovascular diseases.^7^^,^^8^ ANA can also be present in healthy individuals. A population-based study conducted in Japan reported that 26.0% of healthy individuals tested positive for ANA, with significantly higher rates in women.^9^

Similarly, a study conducted in the United States found an ANA prevalence of 13.8% among healthy individuals aged ≥ 12 years, with higher rates observed in women, older adults, and African Americans.^10^ That study also found no significant associations between ANA positivity and the examined factors, including alcohol consumption, smoking status, serum cotinine levels, or C-reactive protein levels. A German population-based study reported an ANA prevalence of 33.3%, particularly among older women, and found no association between ANA positivity and metabolic disorders, such as diabetes, dyslipidemia, obesity, or hypertension.^11^

To date, evidence regarding ANA prevalence in healthy Japanese individuals remains limited. Therefore, this study aimed to evaluate the prevalence of ANA positivity among healthy individuals and to examine the associations between sociodemographic factors and ANA positivity. Understanding ANA prevalence in healthy populations is clinically important because ANA may appear years before the onset of autoimmune disease and may serve as an early predictive biomarker. Establishing population-specific baselines is therefore important for interpreting incidental ANA findings in clinical practice and for designing future longitudinal studies.

## Methods

### Study design

We conducted a cross-sectional study using serum samples collected from community residents in a single prefecture in Japan.

### Participants

Serum samples were obtained from 1,017 residents of a single prefecture in Japan, recruited between June 2014 and April 2019 through annual municipal health examinations and workplace health examinations**.**

### Sampling

Participants were recruited through two parallel streams. The first comprised employees of a single workplace attending a health examination. Of approximately 1,000 eligible employees, 472 consented to participate; the invitation was disseminated through the company, and it could not be confirmed that every eligible employee received it. Five participants who reported a current or past autoimmune disease were excluded, leaving 467 participants aged 20–62 years (46.7% of those eligible). The second comprised 550 residents attending publicly funded health examinations administered by the municipality. Three statutory programs contributed: the Specific Health Checkup for residents aged 40–74 years (n = 492); a health checkup for younger residents (n = 47, aged 20–39 years); and the health checkup for residents aged 75 years and over (n = 11). These sera had been collected for a separate study, and the 550 participants were selected at random from those who had consented to secondary use of their samples, without reference to ANA results or to any participant characteristic. As the denominator of the original collection was not available to us, a participation rate cannot be calculated for this stream. No equivalent exclusion was applied to the 550 participants recruited through municipal programs.

Written informed consent was obtained from all participants. Neither stream constituted a random sample of the general population.

### Variables

Information on age, sex, smoking status, and history of allergic conditions, including hay fever and atopy, was collected using self-administered questionnaires. Smoking status was recorded in three categories (current smoker, former smoker, or never smoker). Because only 11 participants reported former smoking, current and former smokers were combined into a single category for the primary analysis, and a sensitivity analysis restricted to current versus never smokers was also performed. Allergic history was recorded as present or absent. The questionnaire items were not drawn from a formally validated instrument, and allergic history was not confirmed clinically.

### Missing data

The questionnaire covering smoking status and allergic history formed part of the workplace examination only; these items were not collected in any of the municipal programs. Lifestyle data were therefore available only for the 467 participants recruited through workplace examinations and were missing by design for all 550 participants recruited through municipal programs. Because missingness was determined entirely by the administrative program through which a participant was recruited, and because the two streams differ substantially in age distribution (median 36 versus 65 years, with overlap confined to ages 20–62 years), multiple imputation would have required extrapolating the age–lifestyle relationship observed in the younger stream to a substantially older group in which no lifestyle information exists. Because this assumption could not be verified using the available data, complete-case analysis was used, and analyses involving lifestyle factors are explicitly restricted to the workplace subset.

### ANA measurement

ANA positivity was assessed using indirect immunofluorescence (IIF) with HEp-2 cells as the substrate (Fluoro HEPANA Test; MBL, Nagoya, Japan). Serum samples with positive IIF staining at a dilution of 1:40 were classified as ANA-positive in accordance with the manufacturer’s guidelines. Positive and negative control sera supplied with the kit were included in every run, and slides were read according to the manufacturer’s instructions.

According to the manufacturer’s package insert, the assay has a sensitivity of 93.2% in patients with systemic lupus erythematosus and a specificity of 84.1% in normal subjects at a 1:40 dilution; the insert also states that each laboratory should determine its own cutoff value. A threshold of 1:40 was adopted to maximize sensitivity, in line with its established use in routine clinical practice in Japan. Because a low threshold increases the detection of low-titer positivity, the reported prevalence should be interpreted in the context of this cutoff.

Sera were tested as a single qualitative determination at 1:40; endpoint titration was not performed, and individual titers beyond this threshold were therefore unavailable. Fluorescence patterns were not classified according to the International Consensus on ANA Patterns criteria in this study.^12^

### Statistical analysis

All analyses were performed using R version 4.6.0 (R Foundation for Statistical Computing, Vienna, Austria). Two-sided P values below 0.05 were considered statistically significant. The analysis code is provided as Supplementary Material.

Proportions are reported with 95% confidence intervals (CI) calculated by the Wilson score method. ANA positivity was compared between categories using the chi-square test or Fisher’s exact test where cell counts were small. A trend across the six age groups (20–29, 30–39, 40–49, 50–59, 60–69, and ≥70 years) was assessed by the Cochran–Armitage test. Smoking status and allergic history were additionally stratified by sex.

The association between age and ANA positivity was modeled at the individual level by logistic regression including linear and quadratic age terms, fitted separately for the total sample and for each sex. The quadratic term was tested using a likelihood ratio test to assess departure from a monotonic association.

Multivariable logistic regression was performed with sequential adjustment. In the full sample, Model 1 included age and Model 2 added sex. In the subset with complete lifestyle data (n = 467), Model 1′ included age, Model 2′ added sex, and Model 3 further added smoking status and allergic history, so that models differing only in covariates could be compared within the same population. Odds ratios are presented with 95% profile likelihood CIs.

Effect modification of the smoking–ANA association by sex was examined by including a sex × smoking interaction term. Within men, the never-smoker stratum contained only two ANA-positive participants; the age-adjusted association was therefore also estimated using Firth’s penalized likelihood method to reduce small-sample bias.

Two sensitivity analyses were performed. First, the smoking comparison was repeated with the exposed group restricted to current smokers, thereby excluding 11 former smokers. Second, to distinguish the effect of recruitment stream from that of age, ANA positivity was compared between the two streams within their overlapping age range (20–62 years).

To assess whether the missing lifestyle data introduced bias, participants with complete and missing lifestyle data were compared using the Wilcoxon rank-sum test for age and the chi-square test for categorical variables; the results are presented in Table 5.

## Results

A total of 1,017 participants were included in the study, comprising 475 men (46.7%) and 542 women (53.3%). Lifestyle data were unavailable for 550 participants, in whom the relevant questionnaire items were not administered as part of the municipal examination program; analyses involving smoking status and allergic history are therefore restricted to the remaining 467 participants. Table 1 presents the demographic characteristics of the study population, and Table 5 compares participants with complete and missing lifestyle data.

**Table 1:** Basic characteristics of the study population.

Variable	Value
**ANA, n (%)**	**1,017 (100%)**
Positive n (%)	186 (18.3%)
Negative n (%)	831 (81.7%)
**Sex, n (%)**	**1,017 (100%)**
Male n (%)	475 (46.7%)
Female n (%)	542 (53.3%)
**Mean age, years (SD)**	**50.9 (17.1)**
Male y (SD)	51.4 (17.0)
Female y (SD)	50.5 (17.1)
**Age distribution, n (%)**	**1,017 (100%)**
20–29 n (%)	145 (14.3%)
30–39 n (%)	181 (17.8%)
40–49 n (%)	160 (15.7%)
50–59 n (%)	139 (13.7%)
60–69 n (%)	180 (17.7%)
≥70 n (%)	212 (20.8%)
**Smoking status, n (%)**	**467 (100%)**
Never smoker n (%)	272 (58.2%)
Former or current smoker n (%)	195 (41.8%)
**Allergic history, n (%)**	**467 (100%)**
Absent n (%)	335 (71.7%)
Present n (%)	132 (28.3%)

ANA, antinuclear antibodies; SD, standard deviation.

The overall prevalence of ANA positivity was 18.3% (186/1,017; 95% CI 16.0–20.8%). As summarized in Table 2, ANA positivity was significantly higher in women than in men (21.8% vs. 14.3%; P = 0.002) and increased with age (Cochran–Armitage trend test, P < 0.001). In the subset with lifestyle data (n = 467), ANA positivity did not differ significantly by smoking status (12.3% vs. 11.8%; P = 0.86) or allergic history (12.9% vs. 11.6%; P = 0.70).

**Table 2:** Association between basic characteristics and ANA positivity.

Variable	n	ANA-positive, n (%)
**Sex**	**1,017**	**186 (18.3%)**
Male	475	68 (14.3%)
Female	542	118 (21.8%)*
**Age distribution**	**1,017**	**186 (18.3%)**
20–29	145	19 (13.1%)
30–39	181	25 (13.8%)
40–49	160	20 (12.5%)
50–59	139	19 (13.7%)
60–69	180	45 (25.0%)
≥70	212	58 (27.4%)*^a^
**Smoking status**	**467**	**56 (12.0%)**
Never smoker	272	32 (11.8%)
Former or current smoker	195	24 (12.3%)
**Allergic history**	**467**	**56 (12.0%)**
Absent	335	39 (11.6%)
Present	132	17 (12.9%)

⁎ *P* < 0.05. a, Cochran–Armitage trend test. Allergic history included hay fever and atopy. ANA, antinuclear antibodies.

As summarized in Table 3, ANA positivity was higher in women than in men across all age groups, with an overall positivity rate among women of 21.8% (95% CI 18.5–25.4%) compared with 14.3% (95% CI 11.5–17.8%) in men; the difference was statistically significant in the ≥70-year age group (36.8% vs. 17.9%; P = 0.002). In the lifestyle subset (n = 467), ANA positivity was 14.4% (95% CI 10.7–19.1%) in women and 8.9% (95% CI 5.7–13.6%) in men. The association between age and ANA positivity was examined at the individual level using logistic regression with linear and quadratic age terms (Fig. 1). Age was significantly associated with ANA positivity in the total sample (*P* < 0.001) and in both sexes (women, P < 0.001; men, P < 0.001). The quadratic term was significant only in women (P = 0.033), indicating a U-shaped association: predicted positivity declined from 19.9% at age 20 to a nadir of 14.9% at approximately 39 years and rose thereafter to 43.6% at age 78. In men and in the total sample, the quadratic term was not significant (*P* = 0.34 and *P* = 0.24, respectively), indicating a monotonic increase with age.

**Table 3:** Association between basic characteristics and ANA positivity stratified by sex.

Variable	Men		Women		*P* value
	n	ANA-positive, n (%) [95% CI]	n	ANA-positive, n (%) [95% CI]	
**Age distribution (n = 1,017)**	**475**	**68 (14.3%) [11.5–17.8]**	**542**	**118 (21.8%) [18.5–25.4]**	**0.002**
20–29	58	4 (6.9%) [2.7–16.4]	87	15 (17.2%) [10.7–26.5]	0.071
30–39	100	10 (10.0%) [5.5–17.4]	81	15 (18.5%) [11.6–28.3]	0.099
40–49	64	7 (10.9%) [5.4–20.9]	96	13 (13.5%) [8.1–21.8]	0.626
50–59	68	7 (10.3%) [5.1–19.8]	71	12 (16.9%) [9.9–27.3]	0.257
60–69	79	21 (26.6%) [18.1–37.2]	101	24 (23.8%) [16.5–32.9]	0.665
≥70	106	19 (17.9%) [11.8–26.3]	106	39 (36.8%) [28.2–46.3]	0.002
**Lifestyle subset (n = 467)**	**203**	**18 (8.9%) [5.7–13.6]**	**264**	**38 (14.4%) [10.7–19.1]**	**0.068**
Never smoker	83	2 (2.4%) [0.7–8.4]	189	30 (15.9%) [11.4–21.8]	
Former or current smoker	120	16 (13.3%) [8.4–20.6]	75	8 (10.7%) [5.5–19.7]	
Absent	146	14 (9.6%) [5.8–15.5]	189	25 (13.2%) [9.1–18.8]	
Present	57	4 (7.0%) [2.8–16.7]	75	13 (17.3%) [10.4–27.4]	

Values are n (%) with 95% confidence intervals calculated by the Wilson score method. *P* values in the right-hand column compare men and women within each stratum (chi-square test). Within-sex comparisons of smoking status and allergic history were made using Fisher’s exact test: smoking, *P* = 0.010 in men and *P* = 0.334 in women; allergic history, *P* = 0.784 in men and *P* = 0.438 in women. ANA, antinuclear antibodies; CI, confidence interval.

**Fig. 1:**
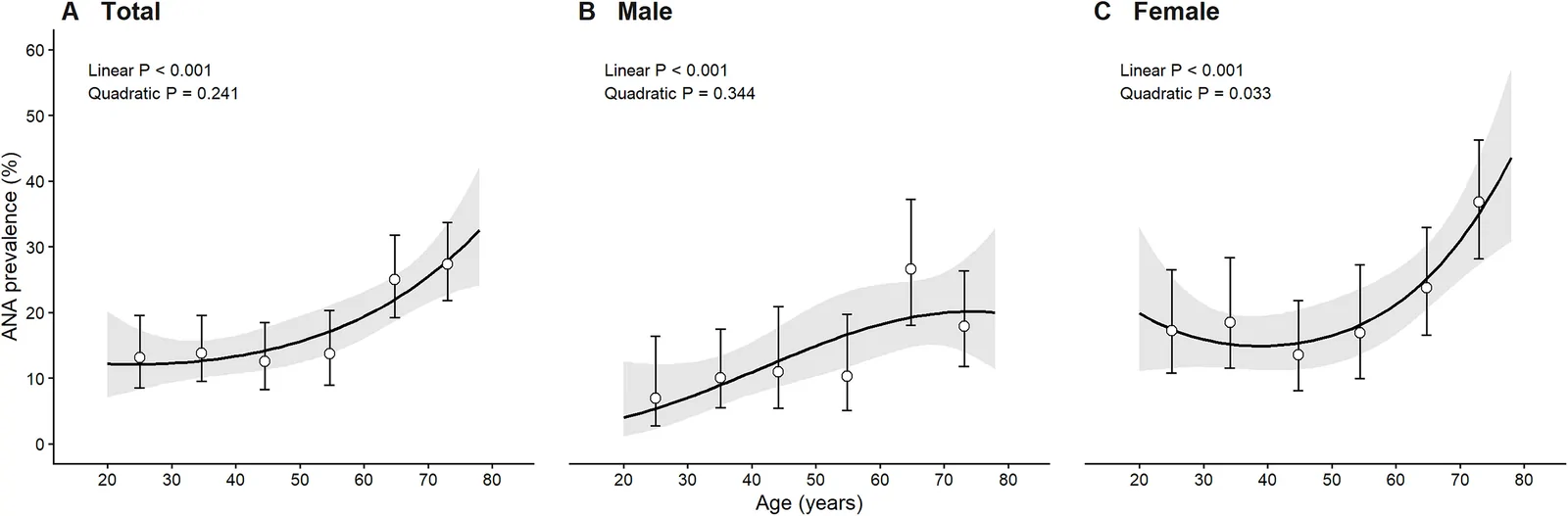
Association between ANA positivity and age, modeled by logistic regression with linear and quadratic age terms. A, total sample (n = 1,017). B, men (n = 475). C, women (n = 542). Solid lines show predicted probabilities with 95% confidence bands (shaded); open circles show observed proportions by age group, plotted at the mean age of each group, with Wilson 95% confidence intervals. P values are from likelihood ratio tests for the linear and quadratic age terms; ANA, antinuclear antibodies.

Among men, current or former smokers had a higher ANA positivity rate than never smokers (13.3% vs. 2.4%; Fisher’s exact test, P = 0.010). This association persisted after adjustment for age (OR 6.27, 95% CI 1.71–40.47), although the never-smoker stratum contained only two ANA-positive participants; using Firth’s penalized likelihood method, the corresponding estimate was OR 5.07 (95% CI 1.31–19.54). The direction was opposite in women (10.7% vs. 15.9%; OR 0.64, 95% CI 0.26–1.40), and the sex × smoking interaction was significant (*P* = 0.003). Restricting the comparison to current versus never smokers, excluding 11 former smokers, gave similar results in men (*P* = 0.009).

There was no evidence of an association between allergic history and ANA positivity, overall or in either sex (men, *P* = 0.78; women, *P* = 0.44), and the sex × allergy interaction was not significant (*P* = 0.34). However, the confidence interval was wide (adjusted OR 1.14, 95% CI 0.60–2.07), and a clinically relevant association cannot be excluded given this sample size.

Multivariable logistic regression was used to assess the associations between ANA positivity and age, sex, smoking status, and allergic history (Table 4). In the full sample, age was independently associated with ANA positivity (Model 1, OR 1.02 per year, 95% CI 1.01–1.03) and remained so after adjustment for sex (Model 2), in which female sex was also independently associated (OR 1.72, 95% CI 1.24–2.40). Within the subset with lifestyle data, age was not associated with ANA positivity even before adjustment for lifestyle factors (Model 1′, OR 1.00, 95% CI 0.98–1.03), indicating that the attenuation of the age effect reflected restriction to this younger subset rather than adjustment for smoking or allergic history. Female sex remained independently associated in the fully adjusted model (Model 3, OR 1.86, 95% CI 1.01–3.55), whereas neither smoking status (OR 1.28, 95% CI 0.70–2.31) nor allergic history (OR 1.14, 95% CI 0.60–2.07) was associated.

**Table 4:** Multivariable logistic regression of factors associated with ANA positivity.

Variable	Model 1 (n = 1,017) OR (95% CI)	Model 1’ (n = 467) OR (95% CI)	Model 2 (n = 1,017) OR (95% CI)	Model 2’ (n = 467) OR (95% CI)	Model 3 (n = 467) OR (95% CI)
**Age**	1.02 (1.01–1.03)	1.00 (0.98–1.03)	1.02 (1.01–1.03)	1.00 (0.98–1.03)	1.00 (0.98–1.03)
**Sex**	—	—	—	—	—
Male	—	—	Reference	Reference	Reference
Female	—	—	1.72 (1.24–2.40)	1.73 (0.97–3.19)	1.86 (1.01–3.55)
**Smoking status**	—	—	—	—	—
Never smoker	—	—	—	—	Reference
Former or current smoker	—	—	—	—	1.28 (0.70–2.31)
**Allergic history**	—	—	—	—	—
Absent	—	—	—	—	Reference
Present	—	—	—	—	1.14 (0.60–2.07)

ANA, antinuclear antibodies; CI, confidence interval; OR, odds ratio; —, variable not included in the model. Odds ratios and confidence intervals are based on profile likelihood. Model 1 and Model 2 were fitted in the full sample (n = 1,017); Models 1′, 2′ and 3 were restricted to participants with complete lifestyle data (n = 467).

To assess potential selection bias, we compared participants with complete lifestyle data (n = 467) and those with missing data (n = 550); Table 5 presents the results. Participants with complete data were significantly younger (median age 36 vs. 65 years; *P* < 0.001) and had significantly lower ANA positivity (12.0% vs. 23.6%; *P* < 0.001), while the sex distribution was similar, with a nonsignificant trend toward a higher proportion of women in the complete-data group (56.5% vs. 50.5%; *P* = 0.057). To distinguish the contribution of age from that of recruitment stream, we repeated this comparison within the age range common to both groups (20–62 years); the difference was substantially attenuated (12.0% vs. 18.6%; *P* = 0.031), indicating that the higher positivity among participants with missing data was largely attributable to their older age.

**Table 5:** Comparison of participants with complete and missing lifestyle data.

Variable	Complete data (n = 467)	Missing data (n = 550)	P value
**Median age, years (IQR)**	36 (28–45)	65 (55–73)	<0.001
**Sex, n (%)**			0.057
Male	203 (43.5%)	272 (49.5%)	
Female	264 (56.5%)	278 (50.5%)	
**ANA positive, n (%)**	56 (12.0%)	130 (23.6%)	<0.001

Age was compared using the Wilcoxon rank-sum test; sex and ANA positivity were compared using the chi-square test. IQR, interquartile range. ANA, antinuclear antibodies.

## Discussion

ANA testing plays an important role in diagnosing autoimmune diseases. However, ANA positivity is not specific to autoimmunity and can also occur as part of nonspecific immune activation.^3^ For example, ANA positivity has been reported in nearly 40% of patients with chronic hepatitis C virus infection due to persistent viral antigenic stimulation that leads to polyclonal B-cell activation.^13^ ANA positivity is frequently observed in healthy individuals.^9^, ^10^, ^11^

Several strengths of this study merit emphasis. First, the relatively large community-based sample of 1,017 participants provides a robust baseline for ANA prevalence estimation in Japan. Second, ANA detection was performed using indirect immunofluorescence on HEp-2 cells, which is the internationally recognized gold standard for ANA testing,^4^^,^^5^^,^^12^ ensuring methodological comparability with prior studies. Third, both sex- and age-stratified analyses were performed, enabling identification of subgroup-specific patterns such as the U-shaped age association in women. Fourth, multivariable logistic regression was performed with sequential adjustment, including models restricted to the subset with complete lifestyle data, so that models differing only in covariates could be compared within the same population.

Reported ANA positivity rates range from 5.92%^14^ to 45.2%^15^ across Europe,^11^^,^^16^^,^^17^ the United States,^10^^,^^18^, ^19^, ^20^ the Middle East,^21^^,^^22^ Oceania,^23^ South America^24^ and Asia,^14^^,^^25^ including several studies from Japan.^9^^,^^15^^,^^26^^,^^27^ Our overall rate of 18.3% lies within this range but is below most previous Japanese IIF-based estimates: 20% among hospital workers in Aichi Prefecture,^26^ 26% among residents of a town in Hyogo Prefecture^9^ and 45.2% in Nagahama City in Shiga Prefecture^15^; it was comparable to the 17.2% reported among pregnant women in Kumamoto Prefecture.^28^ The studies reporting higher prevalence^9^^,^^15^ had older participants and a greater proportion of women, whereas the study reporting comparable prevalence included only pregnant women who were younger than our participants.^28^ Such variation may reflect differences in race and the distributions of age and sex, sample size, testing methodology and cutoff titer, as well as study design, including clinic-based versus community-based sampling and the definition of 'healthy' individuals.

Nevertheless, a consistent finding across many studies^9^, ^10^, ^11^^,^^14^, ^15^, ^16^^,^^19^, ^20^, ^21^^,^^27^ is that ANA positivity is higher among women and older individuals, a pattern also observed in our study. The mechanism underlying the female predominance in ANA positivity is not yet fully understood. However, this observation aligns with extensive evidence showing that women are more likely to develop autoantibodies and autoimmune diseases.^29^ Possible explanations include hormonal influences, particularly estrogen, and incomplete X-chromosome inactivation, which may lead to immune dysregulation.^30^ Estrogen receptors on immune cells may promote B-cell activation and survival, contributing to observed sex differences in ANA prevalence.^30^^,^^31^ Sex hormone levels, genetic differences, and environmental exposures have all been implicated in the onset of autoimmune disease.^31^^,^^32^

In our study, ANA positivity significantly increased with age. However, age was not an independent predictor in the subset with complete lifestyle data. This finding was not attributable to adjustment for lifestyle factors: age was already unassociated with ANA positivity in this subset before smoking status and allergic history were entered into the model (Model 1′, OR 1.00, 95% CI 0.98–1.03). This subset was recruited through workplace examinations and was substantially younger than the full sample (mean age 37.1 vs. 50.9 years, range 20–62 years), so the restricted age distribution rather than confounding likely accounts for the loss of the age effect. Many studies have reported higher ANA prevalence in older adults.^10^^,^^11^^,^^14^, ^15^, ^16^^,^^21^^,^^27^^,^^33^^,^^34^ Higher ANA prevalence in older adults could be related to immunosenescence, whereby age-related immune dysfunction predisposes individuals to increased autoantibody production.^35^^,^^36^

A previous study indicated that the association between ANA positivity and aging in the general population appears to follow a U-shaped pattern, with the lowest ANA levels during the ages of highest reproductive activity.^15^ In our data, this pattern was confirmed in women, in whom the quadratic age term was significant (*P* = 0.033) with a nadir at approximately 39 years, whereas in men and the total sample the association was monotonic (Fig. 1). A similar pattern in women has been reported elsewhere.^9^^,^^11^ However, findings have not been consistent: some have not found higher ANA prevalence in older adults,^18^^,^^20^^,^^24^ and one reported higher ANA frequencies among younger women.^37^ Further work is needed to clarify the relationship between age and ANA prevalence.

We also found that ANA positivity was higher among current and former smokers than among never smokers in men, whereas the direction was opposite in women, and the sex × smoking interaction was statistically significant (*P* = 0.003). A comparable sex-specific effect has been described in rheumatoid arthritis, in which the smoking-related risk is particularly elevated in men^38^ and smoking acts as a sex-specific effect modifier.^39^ Cigarette smoke has broad effects on immunity, inflammation and autoimmunity,^40^^,^^41^ and reactive oxygen species generated by smoking may generate neoantigenic determinants and promote autoantibody formation.^42^^,^^43^ Such mechanisms could plausibly extend to ANA production in healthy individuals. This finding should nevertheless be regarded as exploratory. Only two ANA-positive participants were observed among male never smokers, and the age-adjusted estimate was correspondingly imprecise (OR 5.07, 95% CI 1.31–19.54, Firth’s penalized likelihood method). Previous studies in healthy populations have generally not found strong associations between smoking and ANA positivity,^10^^,^^15^^,^^44^^,^^45^ and although meta-analytic data support an association between current smoking and SLE overall, no significant association has been demonstrated in East Asian populations.^46^ Confirmation in a larger sample with a more balanced distribution of smoking status is required.

We did not identify an association between allergic history and ANA positivity. A comparable observation was reported in a Japanese study of 597 healthy hospital workers, in which the prevalence of atopic dermatitis and allergic rhinitis did not differ significantly between ANA-positive and ANA-negative individuals.^26^ Conversely, ANA—particularly those producing a dense fine speckled pattern—have been reported more frequently in atopic dermatitis and asthma,^24^ suggesting that any association may be confined to specific antibody specificities rather than to ANA positivity overall. Because fluorescence patterns were not classified in the present study, we could not examine this possibility. The confidence interval around our estimate was also wide (adjusted OR 1.14, 95% CI 0.60–2.07), and a clinically relevant association cannot be excluded on the basis of this sample size.

The clinical significance of ANA positivity in apparently healthy individuals remains uncertain. ANA may appear years before the onset of autoimmune disease: ANA were detected before diagnosis in 78% of patients who subsequently developed SLE, at a mean of 3.0 years and up to 9.2 years,^47^ and ANA positivity has been associated with increased long-term risk of autoimmune disease.^16^ Whether the ANA-positive participants identified in this study have a comparable risk cannot be determined from a cross-sectional design, and longitudinal follow-up would be required to establish this.^48^

### Limitations

This study had certain limitations. First, lifestyle information such as body mass index, alcohol consumption, environmental exposures, and full medical history was incomplete or unavailable. Second, conditions that may influence ANA levels, such as active infections, cancer, or current medication use, were not systematically ascertained. Third, autoimmune disease was excluded only in the workplace stream, on the basis of five self-reported cases without clinical verification, so that undiagnosed or unreported cases may remain in either stream and diagnosed cases may be present among the 550 municipal participants. Fourth, ANA was measured only by IIF at a single dilution of 1:40, and endpoint titers were not obtained. This threshold is at the lower end of those commonly used and, while consistent with the manufacturer's guidelines, may yield a higher prevalence than studies using 1:80 or 1:160; because titers were not determined, we could not assess this by sensitivity analysis, and the distribution of titer strength within the ANA-positive group is unknown. Fifth, fluorescence patterns were not classified according to the International Consensus on ANA Patterns criteria, and interobserver agreement was not assessed. Sixth, neither stream constituted a random sample of the general population, and the denominator for the municipal stream was unavailable; therefore, selection bias cannot be excluded and a participation rate cannot be reported for that stream. Seventh, the questionnaire used to ascertain smoking status and allergic history was not a formally validated instrument, and allergic history was based on self-report without clinical confirmation, so misclassification of these exposures is possible. Finally, this study was conducted in a single prefecture, which may limit its representativeness of the general Japanese population. Future studies should include more comprehensive clinical and lifestyle data, as well as additional ANA testing methods, to better evaluate confounding factors and specific ANA subtypes.

## Conclusion

In conclusion, ANA positivity was detected in 18.3% of this Japanese community sample. Female sex was independently associated with positivity, and the association with age differed between the sexes, being monotonic in men and U-shaped in women. These population-specific estimates may assist with the interpretation of incidentally detected ANA in clinical practice, although their prognostic significance remains to be established prospectively.

## Authors' contributions

TT performed the experiments and analyzed the data. YK contributed to data collection. TI contributed to the formal data analysis and advised on the statistical methodology. TH conceived and designed the study and supervised the project. All authors reviewed the manuscript and approved the final draft.

## Ethics declaration

Written informed consent to take part in the study and to publish the article has been obtained from all participants or their legal representatives. The privacy rights of participants have been observed.

This study included organ or tissue donors. This study includes human biological material and consent was obtained by donors, or their next of kin or legal representatives, for use in this study and for publication of the article. The samples used in this research were not sourced from executed prisoners or prisoners of conscience.

This study was performed in compliance with relevant laws, regulatory frameworks and guidelines where the research took place. This study was approved by the Ethics Committee of the University of Miyazaki. (Approval No. 2013-029)

## Ethics approval and consent to participate

This study was approved by the Ethics Committee of the University of Miyazaki [Approval number 2013-029], and written informed consent was obtained from all participants.

## Declaration of generative AI and AI-assisted technologies in the writing process

During the preparation of this work, the authors used Anthropic's Claude to proofread and improve the English phrasing. The authors reviewed and edited the content produced by the tool and take full responsibility for the content of this publication.

## Funding

This work was supported by the Environment Research and Technology Development Fund of the Environmental Restoration and Conservation Agency, Ministry of the Environment of Japan [Grant Number 5-1455].

## Declaration of competing interest

The authors declare that they have no known competing financial interests or personal relationships that could have appeared to influence the work reported in this paper.

The author is an Editorial Board Member/Editor-in-Chief/Associate Editor/Guest Editor for this journal and was not involved in the editorial review or the decision to publish this article.

The authors declare the following financial interests/personal relationships which may be considered as potential competing interests:

## References

[1] Damoiseaux J., Andrade L.E., Fritzler M.J., Shoenfeld Y. (2015). Autoantibodies 2015: from diagnostic biomarkers toward prediction, prognosis and prevention. Autoimmun Rev.

[2] Solomon D.H., Kavanaugh A.J., Schur P.H. (2002). American college of rheumatology Ad Hoc committee on immunologic testing G: evidence-based guidelines for the use of immunologic tests: antinuclear antibody testing. Arthritis Rheum.

[3] Zanussi J.T., Zhao J., Wei W.Q., Karakoc G., Chung C.P., Feng Q., Olsen N.J., Stein C.M., Kawai V.K. (2023). Clinical diagnoses associated with a positive antinuclear antibody test in patients with and without autoimmune disease. BMC Rheumatol.

[4] Kumar Y., Bhatia A., Minz R.W. (2009). Antinuclear antibodies and their detection methods in diagnosis of connective tissue diseases: a journey revisited. Diagn Pathol.

[5] Sur L.M., Floca E., Sur D.G., Colceriu M.C., Samasca G., Sur G. (2018). Antinuclear antibodies: marker of diagnosis and evolution in autoimmune diseases. Lab Med.

[6] Guyomard S., Salles G., Coudurier M., Rousset H., Coiffier B., Bienvenu J., Fabien N. (2003). Prevalence and pattern of antinuclear autoantibodies in 347 patients with non-Hodgkin's lymphoma. Br J Haematol.

[7] Liang K.P., Kremers H.M., Crowson C.S., Snyder M.R., Therneau T.M., Roger V.L., Gabriel S.E. (2009). Autoantibodies and the risk of cardiovascular events. J Rheumatol.

[8] Sedaghat A., Sadeghi M., Heidari R., Sistani E., Bayanfar Z. (2014). Rheumatoid factor, anti-nuclear antibody in ischemic heart disease: acute versus chronic patients. ARYA Atheroscler.

[9] Hayashi N., Koshiba M., Nishimura K., Sugiyama D., Nakamura T., Morinobu S., Kawano S., Kumagai S. (2008). Prevalence of disease-specific antinuclear antibodies in general population: estimates from annual physical examinations of residents of a small town over a 5-year period. Mod Rheumatol.

[10] Satoh M., Chan E.K., Ho L.A., Rose K.M., Parks C.G., Cohn R.D., Jusko T.A., Walker N.J., Germolec D.R., Whitt I.Z. (2012). Prevalence and sociodemographic correlates of antinuclear antibodies in the United States. Arthritis Rheum.

[11] Akmatov M.K., Rober N., Ahrens W., Flesch-Janys D., Fricke J., Greiser H., Gunther K., Kaaks R., Kemmling Y., Krone B. (2017). Anti-nuclear autoantibodies in the general German population: prevalence and lack of association with selected cardiovascular and metabolic disorders-findings of a multicenter population-based study. Arthritis Res Ther.

[12] Tesija Kuna A., Derek L., Drvar V., Kozmar A., Gugo K. (2021). Assessment of antinuclear antibodies (ANA): National recommendations on behalf of the Croatian society of medical biochemistry and laboratory medicine. Biochem Med.

[13] Peng Y.C., Hsieh S.C., Yang D.Y., Tung C.F., Hu W.H., Huang W.N., Chen G.H. (2001). Expression and clinical significance of antinuclear antibody in hepatitis C virus infection. J Clin Gastroenterol.

[14] Guo Y.P., Wang C.G., Liu X., Huang Y.Q., Guo D.L., Jing X.Z., Yuan C.G., Yang S., Liu J.M., Han M.S. (2014). The prevalence of antinuclear antibodies in the general population of china: a cross-sectional study. Curr Ther Res Clin Exp.

[15] Terao C., Ohmura K., Yamada R., Kawaguchi T., Shimizu M., Tabara Y., Takahashi M., Setoh K., Nakayama T., Kosugi S. (2014). Association between antinuclear antibodies and the HLA class II locus and heterogeneous characteristics of staining patterns: the Nagahama study. Arthritis Rheumatol.

[16] Selmi C., Ceribelli A., Generali E., Scire C.A., Alborghetti F., Colloredo G., Porrati L., Achenza M.I., De Santis M., Cavaciocchi F. (2016). Serum antinuclear and extractable nuclear antigen antibody prevalence and associated morbidity and mortality in the general population over 15 years. Autoimmun Rev.

[17] Verstegen G., Duyck M.C., Meeus P., Ravelingien I., De Vlam K. (2009). Detection and identification of antinuclear antibodies (ANA) in a large community hospital. Acta Clin Belg.

[18] Tan E.M., Feltkamp T.E., Smolen J.S., Butcher B., Dawkins R., Fritzler M.J., Gordon T., Hardin J.A., Kalden J.R., Lahita R.G. (1997). Range of antinuclear antibodies in "healthy" individuals. Arthritis Rheum.

[19] Wandstrat A.E., Carr-Johnson F., Branch V., Gray H., Fairhurst A.M., Reimold A., Karp D., Wakeland E.K., Olsen N.J. (2006). Autoantibody profiling to identify individuals at risk for systemic lupus erythematosus. J Autoimmun.

[20] Li Q.Z., Karp D.R., Quan J., Branch V.K., Zhou J., Lian Y., Chong B.F., Wakeland E.K., Olsen N.J. (2011). Risk factors for ANA positivity in healthy persons. Arthritis Res Ther.

[21] Racoubian E., Zubaid R.M., Shareef M.A., Almawi W.Y. (2016). Prevalence of antinuclear antibodies in healthy Lebanese subjects, 2008-2015: a cross-sectional study involving 10,814 subjects. Rheumatol Int.

[22] Shovman O., Gilburd B., Barzilai O., Shinar E., Larida B., Zandman-Goddard G., Binder S.R., Shoenfeld Y. (2005). Evaluation of the BioPlex 2200 ANA screen: analysis of 510 healthy subjects: incidence of natural/predictive autoantibodies. Ann N Y Acad Sci.

[23] Roberts-Thomson P.J., Nikoloutsopoulos T., Cox S., Walker J.G., Gordon T.P. (2003). Antinuclear antibody testing in a regional immunopathology laboratory. Immunol Cell Biol.

[24] Mariz H.A., Sato E.I., Barbosa S.H., Rodrigues S.H., Dellavance A., Andrade L.E. (2011). Pattern on the antinuclear antibody-HEp-2 test is a critical parameter for discriminating antinuclear antibody-positive healthy individuals and patients with autoimmune rheumatic diseases. Arthritis Rheum.

[25] Minz R.W., Kumar Y., Anand S., Singh S., Bamberi P., Verma S., Shegal S. (2012). Antinuclear antibody positive autoimmune disorders in North India: an appraisal. Rheumatol Int.

[26] Watanabe A., Kodera M., Sugiura K., Usuda T., Tan E.M., Takasaki Y., Tomita Y., Muro Y. (2004). Anti-DFS70 antibodies in 597 healthy hospital workers. Arthritis Rheum.

[27] Ishikawa M., Konta T., Hao Z., Takasaki S., Abiko H., Takahashi T., Ikeda A., Ichikawa K., Shibata Y., Takeishi Y. (2008). Relationship between antinuclear antibody and microalbuminuria in the general population: the Takahata study. Clin Exp Nephrol.

[28] Fukushige M., Lu X., Satoh M., Oda M., Ohba T., Katoh T. (2023). Association between antinuclear antibody positivity and chemical exposure among pregnant Japanese women: a cross-sectional study based on the Japan environment and Children's study. Int J Hyg Environ Health.

[29] Youness A., Miquel C.H., Guery J.C. (2021). Escape from X chromosome inactivation and the female predominance in autoimmune diseases. Int J Mol Sci.

[30] Lasrado N., Jia T., Massilamany C., Franco R., Illes Z., Reddy J. (2020). Mechanisms of sex hormones in autoimmunity: focus on EAE. Biol Sex Differ.

[31] Oliver J.E., Silman A.J. (2009). Why are women predisposed to autoimmune rheumatic diseases?. Arthritis Res Ther.

[32] Strickland F.M., Hewagama A., Lu Q., Wu A., Hinderer R., Webb R., Johnson K., Sawalha A.H., Delaney C., Yung R. (2012). Environmental exposure, estrogen and two X chromosomes are required for disease development in an epigenetic model of lupus. J Autoimmun.

[33] Hanarz M., Zabczyk M., Natorska J., Pociask E., Undas A. (2025). Antinuclear antibodies as a risk factor for ischemic stroke or death in elderly patients with atrial fibrillation despite anticoagulation. Clin Rheumatol.

[34] Krzemien P., Kasperczyk S., Banach M., Kasperczyk A., Dobrakowski M., Tomasik T., Windak A., Mastej M., Catapano A., Ray K.K. (2022). Analysis of the impact of sex and age on the variation in the prevalence of antinuclear autoantibodies in Polish population: a nationwide observational, cross-sectional study. Rheumatol Int.

[35] Liang K.P., Gabriel S.E. (2007). Autoantibodies: innocent bystander or key player in immunosenescence and atherosclerosis?. J Rheumatol.

[36] Vadasz Z., Haj T., Kessel A., Toubi E. (2013). Age-related autoimmunity. BMC Med.

[37] Meier H.C.S., Sandler D.P., Simonsick E.M., Weng N.P., Parks C.G. (2020). Sex differences in the association between antinuclear antibody positivity with diabetes and multimorbidity in older adults: results from the Baltimore Longitudinal Study of Aging. Exp Gerontol.

[38] Sugiyama D., Nishimura K., Tamaki K., Tsuji G., Nakazawa T., Morinobu A., Kumagai S. (2010). Impact of smoking as a risk factor for developing rheumatoid arthritis: a meta-analysis of observational studies. Ann Rheum Dis.

[39] Krishnan E., Sokka T., Hannonen P. (2003). Smoking-gender interaction and risk for rheumatoid arthritis. Arthritis Res Ther.

[40] Arnson Y., Shoenfeld Y., Amital H. (2010). Effects of tobacco smoke on immunity, inflammation and autoimmunity. J Autoimmun.

[41] Sopori M.L., Kozak W. (1998). Immunomodulatory effects of cigarette smoke. J Neuroimmunol.

[42] Klareskog L., Malmstrom V., Lundberg K., Padyukov L. (2011). Alfredsson L: Smoking, citrullination and genetic variability in the immunopathogenesis of rheumatoid arthritis. Semin Immunol.

[43] Griffiths H.R. (2008). Is the generation of neo-antigenic determinants by free radicals central to the development of autoimmune rheumatoid disease?. Autoimmun Rev.

[44] Freemer M.M., King T.E., Criswell L.A. (2006). Association of smoking with dsDNA autoantibody production in systemic lupus erythematosus. Ann Rheum Dis.

[45] Young K.A., Terrell D.R., Guthridge J.M., Kamen D.L., Gilkeson G.S., Karp D.R., Ishimori M.L., Weisman M.H., Holers V.M., Harley J.B. (2014). Smoking is not associated with autoantibody production in systemic lupus erythematosus patients, unaffected first-degree relatives, nor healthy controls. Lupus.

[46] Jiang F., Li S., Jia C. (2015). Smoking and the risk of systemic lupus erythematosus: an updated systematic review and cumulative meta-analysis. Clin Rheumatol.

[47] Arbuckle M.R., McClain M.T., Rubertone M.V., Scofield R.H., Dennis G.J., James J.A., Harley J.B. (2003). Development of autoantibodies before the clinical onset of systemic lupus erythematosus. N Engl J Med.

[48] Eriksson C., Kokkonen H., Johansson M., Hallmans G., Wadell G. (2011). Rantapää-Dahlqvist S: Autoantibodies predate the onset of systemic lupus erythematosus in northern Sweden. Arthritis Res Ther.

