# Supplementary material for: Prevalence and correlates of antinuclear antibodies in a Japanese community sample: a cross-sectional study
Source: Curr Ther Res Clin Exp. 2026 Sep 16;105:100845. doi: 10.1016/j.curtheres.2026.100845 (PMC13627905; doi:10.1016/j.curtheres.2026.100845)
Supplement: Supplementary file 1 [file mmc1.pdf]

In [9]:
```
################################################################################
#  CURTHERRES-D-26-00101
#  Prevalence and correlates of antinuclear antibodies in a Japanese
#  community sample: a cross-sectional study
#
#  全統計解析スクリプト（改訂版）
#
#  入力 : Data_Tamanna.xlsx  (sheet "totalall", 1017 rows)
#  出力 : Results_tables.xlsx   全表（Table 1-5 + 追加解析）
#         Figure1_revised.tiff  投稿用図（300 dpi, LZW）
#         analysis_log.txt      実行ログ（照合用）
#
#  必要パッケージ:
#      install.packages(c("openxlsx", "brglm2", "ggplot2"))
#      ※ Firth法は brglm2 を使用。logistf でも可（下記 B2 参照）
#
#  注意:
#   - ロジスティック回帰の信頼区間は confint() = プロファイル尤度に基づく。
#     confint.default() は Wald 法なので使用しない（JMPの既定と一致させるため）
#   - 変数 antibody は 1=陽性 / 0=陰性
#   - 変数 group は c=職域健診(n=467) / s=市町村健診(n=550)
################################################################################
```

In [1]:
```
library(openxlsx)
library(brglm2)
library(ggplot2)
```

```
Warning message:
"package 'brglm2' was built under R version 4.6.1"
```

In [2]:
```
IN_XLSX  <- "D:/OneDrive/OneDrive - 滋賀大学/Miyazaki_University/指導/TAMANNNA/TA
OUT_XLSX <- "Results_tables.xlsx"
OUT_TIFF <- "Figure1_revised.tiff"
OUT_LOG  <- "analysis_log.txt"
```

In [3]:
```
sink(OUT_LOG, split = TRUE)   # 画面とファイルの両方に出力
cat("=== CURTHERRES-D-26-00101  統計解析ログ ===\n")
cat("実行日時 :", format(Sys.time(), "%Y-%m-%d %H:%M:%S"), "\n")
cat("R version:", R.version.string, "\n\n")
```

```
=== CURTHERRES-D-26-00101  統計解析ログ ===
実行日時 : 2026-07-25 00:12:44
R version: R version 4.6.0 (2026-04-24 ucrt)
```

In [4]:
```
## =============================================================================
## 0. データ読み込みと派生変数
## =============================================================================
d <- openxlsx::read.xlsx(IN_XLSX, sheet = "totalall")
```

In [5]:
```
stopifnot(nrow(d) == 1017)
```

In [6]:
```
d$female <- as.integer(d$sex == "f")
d$pos    <- d$antibody                         # 1 = ANA positive
```

In [7]:
```
# 喫煙: never / ever (current + past)。欠測は NA のまま
d$ever <- ifelse(is.na(d$smoke), NA,
```

```
           ifelse(d$smoke == "never", "never", "ever"))
d$ever <- factor(d$ever, levels = c("never", "ever"))

# 現喫煙のみ（感度解析B3a用: past を除外）
d$cur_only <- ifelse(is.na(d$smoke), NA,
              ifelse(d$smoke == "never", "never",
              ifelse(d$smoke == "current", "current", NA)))
d$cur_only <- factor(d$cur_only, levels = c("never", "current"))
```

In [8]:
```
# アレルギー
d$allergy <- ifelse(is.na(d$illness), NA,
             ifelse(d$illness == "allergy", "with", "without"))
d$allergy <- factor(d$allergy, levels = c("without", "with"))

d$sexf <- factor(d$sex, levels = c("m", "f"))
```

In [9]:
```
cc <- subset(d, group == "c")                # 完全データ集団 (n=467)
ms <- subset(d, group == "s")                # 欠測集団 (n=550)

cat("読み込み確認: n =", nrow(d),
    " / group c =", nrow(cc), " / group s =", nrow(ms), "\n\n")
```

```
読み込み確認: n = 1017  / group c = 467  / group s = 550
```

In [10]:
```
## --- 補助関数 -------------------------------------------------------------
# Wilson スコア信頼区間（base R の prop.test, 連続修正なし）
wilson <- function(x, n) {
  ci <- prop.test(x, n, correct = FALSE)$conf.int
  c(pct = 100 * x / n, lo = 100 * ci[1], hi = 100 * ci[2])
}
# 「陽性数 (割合%) [95%CI]」の整形
fmt_prev <- function(x, n) {
  w <- wilson(x, n)
  sprintf("%d/%d (%.1f) [%.1f-%.1f]", x, n, w["pct"], w["lo"], w["hi"])
}
# glm からオッズ比・プロファイル尤度CI・尤度比検定P値を取り出す
#   CIとP値をいずれも尤度に基づく方法で統一する（JMPの既定と同じ）
#   ※ Wald法（summary()のP値, confint.default()）とは僅かに異なる。
#      例: Model 3 の sex は Wald p=0.051 だが尤度比 p<0.05。CIが1を跨がない
#          ことと整合するのは尤度比検定のほう。
or_table <- function(fit, model_name) {
  co <- summary(fit)$coefficients
  ci <- suppressMessages(confint(fit))            # プロファイル尤度CI
  lr <- drop1(fit, test = "LRT")                  # 尤度比検定
  tm <- rownames(co); keep <- tm != "(Intercept)"

  # 係数名 → 説明変数名 の対応（因子は "sexff" → "sexf" のように解決する）
  vars <- rownames(lr)[-1]
  match_var <- function(term) vars[which.max(nchar(vars[sapply(vars,
               function(v) startsWith(term, v))]))]
  p_lr <- sapply(tm[keep], function(t) {
    v <- vars[sapply(vars, function(x) startsWith(t, x))]
    if (length(v) == 0) NA else lr[v[which.max(nchar(v))], "Pr(>Chi)"]
  })

  data.frame(
    Model    = model_name,
    Term     = tm[keep],
    OR       = round(exp(co[keep, 1]), 3),
```

```
    CI_low  = round(exp(ci[keep, 1]), 3),
    CI_high = round(exp(ci[keep, 2]), 3),
    P_LR    = signif(p_lr, 3),
    P_Wald  = signif(co[keep, 4], 3),
    row.names = NULL
  )
}
```

In [11]:

```
################################################################################
## 1. Table 1  基本特性
################################################################################
cat("\n########## Table 1: Basic characteristics ##########\n")
t1 <- rbind(
  data.frame(Variable = "ANA positive", Category = "-",
             n = sum(d$pos), Pct = round(100 * mean(d$pos), 1)),
  data.frame(Variable = "Sex", Category = c("Male", "Female"),
             n = c(sum(d$sex == "m"), sum(d$sex == "f")),
             Pct = round(100 * c(mean(d$sex == "m"), mean(d$sex == "f")), 1)),
  data.frame(Variable = "Age group",
             Category = c("20-29","30-39","40-49","50-59","60-69","70+"),
             n = as.vector(table(d$age_generation)),
             Pct = round(100 * as.vector(prop.table(table(d$age_generation))), 1
  data.frame(Variable = "Smoking (n=467)", Category = c("Never", "Former/current
             n = as.vector(table(cc$ever)),
             Pct = round(100 * as.vector(prop.table(table(cc$ever))), 1)),
  data.frame(Variable = "Allergy (n=467)", Category = c("Without", "With"),
             n = as.vector(table(cc$allergy)),
             Pct = round(100 * as.vector(prop.table(table(cc$allergy))), 1))
)
print(t1, row.names = FALSE)
cat("\n平均年齢 (SD): 全体", sprintf("%.1f (%.1f)", mean(d$age), sd(d$age)),
    "/ 男", sprintf("%.1f (%.1f)", mean(d$age[d$sex=="m"]), sd(d$age[d$sex=="m"]
    "/ 女", sprintf("%.1f (%.1f)", mean(d$age[d$sex=="f"]), sd(d$age[d$sex=="f"]
```

```
########## Table 1: Basic characteristics ##########
        Variable       Category   n  Pct
    ANA positive              - 186 18.3
             Sex           Male 475 46.7
             Sex         Female 542 53.3
       Age group          20-29 145 14.3
       Age group          30-39 181 17.8
       Age group          40-49 160 15.7
       Age group          50-59 139 13.7
       Age group          60-69 180 17.7
       Age group            70+ 212 20.8
 Smoking (n=467)          Never 272 58.2
 Smoking (n=467) Former/current 195 41.8
 Allergy (n=467)        Without 335 71.7
 Allergy (n=467)           With 132 28.3

平均年齢 (SD): 全体 50.9 (17.1) / 男 51.4 (17.0) / 女 50.5 (17.1)
```

In [13]:

```
################################################################################
## 2. Table 2  基本特性別 ANA陽性率  (+ Cochran-Armitage 傾向検定)
################################################################################
cat("\n########## Table 2: ANA positivity by characteristic ##########\n")
mk2 <- function(varname, lv, sub) {
  do.call(rbind, lapply(lv, function(l) {
    s <- sub[[1]][sub[[2]] == l]
    data.frame(Variable = varname, Category = as.character(l),
```

```
                    Result = fmt_prev(sum(s), length(s)))
  }))
}
t2 <- rbind(
  data.frame(Variable = "Overall", Category = "-",
             Result = fmt_prev(sum(d$pos), nrow(d))),
  mk2("Sex", c("m","f"), list(d$pos, d$sex)),
  mk2("Age group", sort(unique(d$age_generation)), list(d$pos, d$age_generation)
  mk2("Smoking (n=467)", c("never","ever"), list(cc$pos, cc$ever)),
  mk2("Allergy (n=467)", c("without","with"), list(cc$pos, cc$allergy))
)
print(t2, row.names = FALSE)

cat("\n-- 検定 --\n")
p_sex <- chisq.test(table(d$sex, d$pos), correct = FALSE)
cat("性別 (chi-square, uncorrected): p =", signif(p_sex$p.value, 4), "\n")

tabg <- table(d$age_generation, d$pos)
ca <- prop.trend.test(tabg[, "1"], rowSums(tabg))
cat("年齢 Cochran-Armitage trend test: X2 =", round(ca$statistic, 4),
    ", df =", ca$parameter, ", p =", signif(ca$p.value, 5), "\n")
cat("喫煙 (chi-square):", signif(chisq.test(table(cc$ever, cc$pos),
                                   correct = FALSE)$p.value, 4), "\n")
cat("アレルギー (chi-square):", signif(chisq.test(table(cc$allergy, cc$pos),
                                   correct = FALSE)$p.value, 4), "\n")
```

```
########## Table 2: ANA positivity by characteristic ##########
        Variable Category                        Result
         Overall        - 186/1017 (18.3) [16.0-20.8]
             Sex        m   68/475 (14.3) [11.5-17.8]
             Sex        f  118/542 (21.8) [18.5-25.4]
       Age group       20    19/145 (13.1) [8.6-19.6]
       Age group       30    25/181 (13.8) [9.5-19.6]
       Age group       40    20/160 (12.5) [8.2-18.5]
       Age group       50    19/139 (13.7) [8.9-20.4]
       Age group       60   45/180 (25.0) [19.2-31.8]
       Age group       70   58/212 (27.4) [21.8-33.7]
 Smoking (n=467)    never    32/272 (11.8) [8.5-16.1]
 Smoking (n=467)     ever    24/195 (12.3) [8.4-17.7]
 Allergy (n=467)  without    39/335 (11.6) [8.6-15.5]
 Allergy (n=467)     with    17/132 (12.9) [8.2-19.7]

-- 検定 --
性別 (chi-square, uncorrected): p = 0.002152
年齢 Cochran-Armitage trend test: X2 = 21.0062 , df = 1 , p = 4.5781e-06

-- 検定 --
性別 (chi-square, uncorrected): p = 0.002152
年齢 Cochran-Armitage trend test: X2 = 21.0062 , df = 1 , p = 4.5781e-06
喫煙 (chi-square): 0.8586
アレルギー (chi-square): 0.711
```

In [14]:

```
################################################################################
## 3. Table 3  性別層別  (男性喫煙は Fisher 正確検定)
################################################################################
cat("\n########## Table 3: Stratified by sex ##########\n")
t3 <- do.call(rbind, lapply(c("m", "f"), function(sx) {
  ds <- subset(d,  sex == sx)
  dc <- subset(cc, sex == sx)
  rbind(
```

```
    do.call(rbind, lapply(sort(unique(ds$age_generation)), function(g) {
      s <- ds$pos[ds$age_generation == g]
      data.frame(Sex = sx, Variable = "Age group", Category = as.character(g),
                 Result = fmt_prev(sum(s), length(s)))
    })),
    do.call(rbind, lapply(c("never","ever"), function(l) {
      s <- dc$pos[dc$ever == l]
      data.frame(Sex = sx, Variable = "Smoking", Category = l,
                 Result = fmt_prev(sum(s), length(s)))
    })),
    do.call(rbind, lapply(c("without","with"), function(l) {
      s <- dc$pos[dc$allergy == l]
      data.frame(Sex = sx, Variable = "Allergy", Category = l,
                 Result = fmt_prev(sum(s), length(s)))
    }))
  )
}))
print(t3, row.names = FALSE)

cat("\n-- 喫煙とANAの関連（性別） --\n")
for (sx in c("m", "f")) {
  tb <- table(subset(cc, sex == sx)$ever, subset(cc, sex == sx)$pos)
  cat(sprintf("%s: chi2 p = %.4f / Fisher exact p = %.4f\n", sx,
              chisq.test(tb, correct = FALSE)$p.value,
              fisher.test(tb)$p.value))
}
cat("  ※ 男性 never smoker はイベント2件のため Fisher を採用する\n")
```

```
########## Table 3: Stratified by sex ##########
 Sex  Variable Category                      Result
   m Age group       20      4/58 (6.9) [2.7-16.4]
   m Age group       30   10/100 (10.0) [5.5-17.4]
   m Age group       40     7/64 (10.9) [5.4-20.9]
   m Age group       50     7/68 (10.3) [5.1-19.8]
   m Age group       60   21/79 (26.6) [18.1-37.2]
   m Age group       70 19/106 (17.9) [11.8-26.3]
   m   Smoking    never       2/83 (2.4) [0.7-8.4]
   m   Smoking     ever   16/120 (13.3) [8.4-20.6]
   m   Allergy  without    14/146 (9.6) [5.8-15.5]
   m   Allergy     with      4/57 (7.0) [2.8-16.7]
   f Age group       20   15/87 (17.2) [10.7-26.5]
   f Age group       30   15/81 (18.5) [11.6-28.3]
   f Age group       40    13/96 (13.5) [8.1-21.8]
   f Age group       50    12/71 (16.9) [9.9-27.3]
   f Age group       60 24/101 (23.8) [16.5-32.9]
   f Age group       70 39/106 (36.8) [28.2-46.3]
   f   Smoking    never 30/189 (15.9) [11.4-21.8]
   f   Smoking     ever     8/75 (10.7) [5.5-19.7]
   f   Allergy  without  25/189 (13.2) [9.1-18.8]
   f   Allergy     with  13/75 (17.3) [10.4-27.4]

-- 喫煙とANAの関連（性別） --
m: chi2 p = 0.0071 / Fisher exact p = 0.0101
f: chi2 p = 0.2771 / Fisher exact p = 0.3341
  ※ 男性 never smoker はイベント2件のため Fisher を採用する
```

In [16]:
```
################################################################################
## 4. Table 4  ロジスティック回帰 Model 1, 2, 1', 2', 3
################################################################################
cat("\n########## Table 4: Logistic regression ##########\n")
```

```
m1  <- glm(pos ~ age,                          data = d,  family = binomial)
m2  <- glm(pos ~ age + sexf,                   data = d,  family = binomial)
m1p <- glm(pos ~ age,                          data = cc, family = binomial)
m2p <- glm(pos ~ age + sexf,                   data = cc, family = binomial)
m3  <- glm(pos ~ age + sexf + ever + allergy,  data = cc, family = binomial)

t4 <- rbind(
  or_table(m1,  "Model 1  (n=1017)"),
  or_table(m2,  "Model 2  (n=1017)"),
  or_table(m1p, "Model 1' (n=467)"),
  or_table(m2p, "Model 2' (n=467)"),
  or_table(m3,  "Model 3  (n=467)")
)
print(t4, row.names = FALSE)
cat("\n※ 信頼区間はプロファイル尤度に基づく（JMPの既定と一致）\n")
cat("※ Model 1' で既に age は非有意 → 年齢効果の消失は共変量調整ではなく\n",
    "  n=467 への限定によることを示す\n")
```

```
########## Table 4: Logistic regression ##########
             Model        Term    OR CI_low CI_high     P_LR   P_Wald
 Model 1  (n=1017)         age 1.023  1.014   1.034 2.14e-06 3.82e-06
 Model 2  (n=1017)         age 1.024  1.014   1.034 1.30e-06 2.44e-06
 Model 2  (n=1017)       sexff 1.718  1.237   2.404 1.20e-03 1.39e-03
  Model 1' (n=467)         age 1.005  0.978   1.032 7.21e-01 7.21e-01
  Model 2' (n=467)         age 1.005  0.978   1.032 7.34e-01 7.34e-01
  Model 2' (n=467)       sexff 1.727  0.967   3.190 6.53e-02 7.13e-02
  Model 3  (n=467)         age 1.004  0.978   1.031 7.52e-01 7.52e-01
  Model 3  (n=467)       sexff 1.863  1.011   3.547 4.59e-02 5.08e-02
  Model 3  (n=467)    everever 1.275  0.697   2.313 4.27e-01 4.26e-01
  Model 3  (n=467) allergywith 1.139  0.604   2.072 6.80e-01 6.78e-01

※ 信頼区間はプロファイル尤度に基づく（JMPの既定と一致）
※ Model 1' で既に age は非有意 → 年齢効果の消失は共変量調整ではなく
   n=467 への限定によることを示す
```


```
※ 信頼区間はプロファイル尤度に基づく（JMPの既定と一致）
※ Model 1' で既に age は非有意 → 年齢効果の消失は共変量調整ではなく
   n=467 への限定によることを示す
```


In [18]:

```
################################################################################
## 5. Table 5  完全データ群 vs 欠測群
################################################################################
cat("\n########## Table 5: Complete vs missing lifestyle data ##########\n")
wt <- wilcox.test(cc$age, ms$age)
cat(sprintf("年齢: 完全 %.1f (SD %.1f) vs 欠測 %.1f (SD %.1f), Wilcoxon p = %.3g\
            mean(cc$age), sd(cc$age), mean(ms$age), sd(ms$age), wt$p.value))
cat(sprintf("  参考 Welch t-test p = %.3g\n", t.test(cc$age, ms$age)$p.value))
tsex <- table(d$group, d$sex)
cat(sprintf("性別: 完全 男%d/女%d vs 欠測 男%d/女%d, chi-square p = %.4f\n",
            tsex["c","m"], tsex["c","f"], tsex["s","m"], tsex["s","f"],
            chisq.test(tsex, correct = FALSE)$p.value))
tana <- table(d$group, d$pos)
cat(sprintf("ANA陽性: 完全 %s vs 欠測 %s, chi-square p = %.3g\n",
            fmt_prev(sum(cc$pos), nrow(cc)), fmt_prev(sum(ms$pos), nrow(ms)),
            chisq.test(tana, correct = FALSE)$p.value))
```

```
########## Table 5: Complete vs missing lifestyle data ##########
年齢: 完全 37.1 (SD 10.4) vs 欠測 62.6 (SD 12.2), Wilcoxon p = 1.87e-121
  参考 Welch t-test p = 6.97e-184
性別: 完全 男203/女264 vs 欠測 男272/女278, chi-square p = 0.0566
ANA陽性: 完全 56/467 (12.0) [9.4-15.3] vs 欠測 130/550 (23.6) [20.3-27.4], chi-squ
are p = 1.69e-06
```


```
  参考 Welch t-test p = 6.97e-184
性別: 完全 男203/女264 vs 欠測 男272/女278, chi-square p = 0.0566
ANA陽性: 完全 56/467 (12.0) [9.4-15.3] vs 欠測 130/550 (23.6) [20.3-27.4], chi-squ
are p = 1.69e-06
```


In [20]:

```r
################################################################################
## 6. Figure 1  年齢との関連（個票レベル 2次ロジスティック）
################################################################################
cat("\n########### Figure 1: Age association (individual level) ##########\n")
grps <- list(Total = d, Male = subset(d, sex == "m"), Female = subset(d, sex ==
ages <- 20:78
pred_all <- NULL; fit_all <- NULL

for (nm in names(grps)) {
  g   <- grps[[nm]]
  ctr <- mean(g$age)
  g$a  <- g$age
  g$a2 <- (g$age - ctr)^2

  full  <- glm(pos ~ a + a2, data = g, family = binomial)
  no_a  <- glm(pos ~ a2,     data = g, family = binomial)
  no_a2 <- glm(pos ~ a,      data = g, family = binomial)

  # 尤度比検定（JMPの「効果に対する尤度比検定」と同じ定義）
  lr  <- function(f0, f1) {
    x <- 2 * (logLik(f1) - logLik(f0))
    c(chi2 = as.numeric(x), p = pchisq(as.numeric(x), 1, lower.tail = FALSE))
  }
  L <- lr(no_a, full); Q <- lr(no_a2, full)

  nd  <- data.frame(a = ages, a2 = (ages - ctr)^2)
  pr  <- predict(full, newdata = nd, type = "link", se.fit = TRUE)
  inv <- function(z) 100 / (1 + exp(-z))

  pred_all <- rbind(pred_all, data.frame(
    Group = nm, Age = ages,
    Predicted = round(inv(pr$fit), 2),
    Lower95   = round(inv(pr$fit - 1.96 * pr$se.fit), 2),
    Upper95   = round(inv(pr$fit + 1.96 * pr$se.fit), 2)))

  b <- coef(full)
  nadir <- if (b["a2"] > 0 && Q["p"] < 0.05) ctr - b["a"] / (2 * b["a2"]) else N

  fit_all <- rbind(fit_all, data.frame(
    Group = nm, n = nrow(g), mean_age = round(ctr, 4),
    b0 = round(b[1], 7), b1_age = round(b["a"], 7), b2_age2 = round(b["a2"], 9),
    LR_chi2_linear = round(L["chi2"], 4), P_linear = signif(L["p"], 3),
    LR_chi2_quad   = round(Q["chi2"], 4), P_quadratic = signif(Q["p"], 3),
    Nadir_age = if (is.na(nadir)) NA else round(nadir, 1), row.names = NULL))
}
print(fit_all, row.names = FALSE)
cat("\n※ JMP出力と一致\n")

# 実測点（年齢群別 + Wilson CI）
```

```
obs_all <- do.call(rbind, lapply(names(grps), function(nm) {
  g <- grps[[nm]]
  do.call(rbind, lapply(sort(unique(g$age_generation)), function(gg) {
    s <- g[g$age_generation == gg, ]
    w <- wilson(sum(s$pos), nrow(s))
    data.frame(Group = nm, Age_group = gg, Age = round(mean(s$age), 2),
               N = nrow(s), Positive = sum(s$pos),
               Observed = round(w["pct"], 2),
               Obs_Lower = round(w["lo"], 2), Obs_Upper = round(w["hi"], 2),
               row.names = NULL)
  }))
}))
```

```
########## Figure 1: Age association (individual level) ##########
  Group    n mean_age        b0    b1_age      b2_age2 LR_chi2_linear P_linear
  Total 1017  50.9105 -2.846573 0.0231041  0.000428883        23.2066 1.45e-06
   Male  475  51.3600 -3.147673 0.0281445 -0.000609682        11.2797 7.84e-04
 Female  542  50.5166 -2.751306 0.0225502  0.000973474        14.0714 1.76e-04
 LR_chi2_quad P_quadratic Nadir_age
       1.3720      0.2410        NA
       0.8947      0.3440        NA
       4.5492      0.0329      38.9
```

※ JMP出力と一致

※ JMP出力と一致

In [21]:

```
# --- 作図 ---------------------------------------------------------------
lab <- c(Total = "A   Total", Male = "B   Male", Female = "C   Female")
pred_all$Panel <- factor(lab[pred_all$Group], levels = lab)
obs_all$Panel  <- factor(lab[obs_all$Group],  levels = lab)
ann <- data.frame(
  Panel = factor(lab[fit_all$Group], levels = lab),
  txt = sprintf("Linear %s\nQuadratic %s",
        ifelse(fit_all$P_linear    < 0.001, "P < 0.001", sprintf("P = %.3f", fit
        ifelse(fit_all$P_quadratic < 0.001, "P < 0.001", sprintf("P = %.3f", fit

p <- ggplot() +
  geom_ribbon(data = pred_all, aes(Age, ymin = Lower95, ymax = Upper95),
              fill = "grey80", alpha = .55) +
  geom_line(data = pred_all, aes(Age, Predicted), linewidth = .6) +
  geom_errorbar(data = obs_all, aes(Age, ymin = Obs_Lower, ymax = Obs_Upper),
                width = 1.6, linewidth = .35) +
  geom_point(data = obs_all, aes(Age, Observed), shape = 21,
             fill = "white", size = 1.9, stroke = .5) +
  geom_text(data = ann, aes(x = 17, y = 58, label = txt),
            hjust = 0, vjust = 1, size = 2.6, lineheight = 1.15) +
  facet_wrap(~ Panel) +
  scale_x_continuous(limits = c(15, 83), breaks = seq(20, 80, 10)) +
  scale_y_continuous(limits = c(0, 60), breaks = seq(0, 60, 10)) +
  labs(x = "Age (years)", y = "ANA prevalence (%)") +
  theme_classic(base_size = 9) +
  theme(strip.background = element_blank(),
        strip.text = element_text(hjust = 0, face = "bold", size = 11),
        panel.spacing = unit(1.1, "lines"))

tiff(OUT_TIFF, width = 9.6, height = 3.3, units = "in", res = 300,
     compression = "lzw")
print(p)
```

```
invisible(dev.off())
cat("図を出力しました:", OUT_TIFF, "\n")
```

図を出力しました: Figure1_revised.tiff

In [23]:

```
################################################################################
## 7. B1  性 × 喫煙 の交互作用
################################################################################
cat("\n########## B1: Sex x smoking interaction ##########\n")
i0 <- glm(pos ~ age + sexf + ever,        data = cc, family = binomial)
i1 <- glm(pos ~ age + sexf * ever,        data = cc, family = binomial)
an <- anova(i0, i1, test = "LRT")
cat("交互作用 sexf:ever\n")
cat("  尤度比検定 p =", signif(an$`Pr(>Chi)`[2], 4), " ← 報告用\n")
cat("  Wald検定   p =", signif(summary(i1)$coefficients["sexff:everever", 4], 4)
print(round(summary(i1)$coefficients, 4))
```

```
########## B1: Sex x smoking interaction ##########
交互作用 sexf:ever
  尤度比検定 p = 0.002515  ← 報告用
  Wald検定   p = 0.008805

  尤度比検定 p = 0.002515  ← 報告用
  Wald検定   p = 0.008805

               Estimate Std. Error z value Pr(>|z|)
(Intercept)     -3.8772     0.8820 -4.3957   0.0000
age              0.0048     0.0139  0.3439   0.7309
sexff            2.0329     0.7430  2.7361   0.0062
everever         1.8272     0.7645  2.3900   0.0169
sexff:everever  -2.2900     0.8742 -2.6196   0.0088
```

In [24]:

```
################################################################################
## 8. B2  男性内・年齢調整した喫煙効果（通常ML と Firth）
################################################################################
cat("\n########## B2: Smoking effect within males (age-adjusted) ##########\n")
cm <- subset(cc, sex == "m")
cat("男性 never smoker のイベント数 =", sum(cm$pos[cm$ever == "never"]), "\n")

ml <- glm(pos ~ ever + age, data = cm, family = binomial)
cat("\n-- 通常の最尤法 --\n")
print(or_table(ml, "Male ML"))

# Firth（brglm2）。logistf を使う場合:  logistf::logistf(pos ~ ever + age, data=cı
fh <- glm(pos ~ ever + age, data = cm, family = binomial,
          method = "brglmFit", type = "AS_mean")
cf <- summary(fh)$coefficients
cat("\n-- Firth 罰則付き最尤法 (brglm2) --\n")
print(data.frame(
  Term = rownames(cf)[-1],
  OR      = round(exp(cf[-1, 1]), 3),
  CI_low  = round(exp(cf[-1, 1] - 1.96 * cf[-1, 2]), 3),
  CI_high = round(exp(cf[-1, 1] + 1.96 * cf[-1, 2]), 3),
  P       = signif(cf[-1, 4], 3), row.names = NULL))
```

```
########## B2: Smoking effect within males (age-adjusted) ##########
男性 never smoker のイベント数 = 2

-- 通常の最尤法 --
男性 never smoker のイベント数 = 2

-- 通常の最尤法 --
    Model      Term    OR CI_low CI_high     P_LR P_Wald
1 Male ML everever 6.268  1.711  40.473 0.00359 0.0166
2 Male ML      age 1.028  0.976   1.083 0.29200 0.2910

-- Firth 罰則付き最尤法 (brglm2) --
      Term    OR CI_low CI_high      P
1 everever 5.067  1.314  19.543 0.0185
2      age 1.028  0.978   1.080 0.2790
```

In [25]:

```
############################################################################
## 9. B3a  感度解析: past smoker を除外 (current vs never)
############################################################################
cat("\n########## B3a: Sensitivity - current vs never (past excluded) ##########
for (sx in c("m", "f")) {
  s <- subset(cc, sex == sx & !is.na(cur_only))
  tb <- table(s$cur_only, s$pos)
  cat(sprintf("%s: never %d/%d, current %d/%d, Fisher p = %.4f\n", sx,
              tb["never","1"], sum(tb["never",]),
              tb["current","1"], sum(tb["current",]),
              fisher.test(tb)$p.value))
}
```

```
########## B3a: Sensitivity - current vs never (past excluded) ##########
m: never 2/83, current 15/114, Fisher p = 0.0089
f: never 30/189, current 7/70, Fisher p = 0.3172
m: never 2/83, current 15/114, Fisher p = 0.0089
f: never 30/189, current 7/70, Fisher p = 0.3172
```

In [26]:

```
############################################################################
## 10. B3b  感度解析: 共通年齢域(20-62歳)での群間比較   ★選択バイアス評価
############################################################################
cat("\n########## B3b: Sensitivity - overlapping age range ##########\n")
cat(sprintf("group c の年齢範囲: %d-%d / group s: %d-%d\n",
            min(cc$age), max(cc$age), min(ms$age), max(ms$age)))
ov <- subset(d, age >= 20 & age <= 62)
tb <- table(ov$group, ov$pos)
cat(sprintf("20-62歳に限定: c %s vs s %s, chi-square p = %.4f\n",
            fmt_prev(tb["c","1"], sum(tb["c",])),
            fmt_prev(tb["s","1"], sum(tb["s",])),
            chisq.test(tb, correct = FALSE)$p.value))
cat(sprintf(" (参考) 全年齢: c %s vs s %s, p = %.3g\n",
            fmt_prev(sum(cc$pos), nrow(cc)), fmt_prev(sum(ms$pos), nrow(ms)),
            chisq.test(table(d$group, d$pos), correct = FALSE)$p.value))
cat("\n※ 年齢を揃えると群間差が縮小 → 欠測群のANA高値は主に年齢交絡による\n")
```

```
########## B3b: Sensitivity - overlapping age range ##########
group c の年齢範囲: 20-62 / group s: 20-78
20-62歳に限定: c 56/467 (12.0) [9.4-15.3] vs s 32/172 (18.6) [13.5-25.1], chi-square p = 0.0314
 (参考) 全年齢: c 56/467 (12.0) [9.4-15.3] vs s 130/550 (23.6) [20.3-27.4], p = 1.69e-06

※ 年齢を揃えると群間差が縮小 → 欠測群のANA高値は主に年齢交絡による
```

In [27]:
```
##############################################################################
## 11. B4  有病率の Wilson 95%信頼区間（R#4: CIを一貫して報告）
##############################################################################
cat("\n########## B4: Prevalence with Wilson 95% CI ##########\n")
b4 <- rbind(
  data.frame(Group = "Overall", Category = "-", Result = fmt_prev(sum(d$pos), nr
  do.call(rbind, lapply(c("m","f"), function(s)
    data.frame(Group = "Sex", Category = s,
               Result = fmt_prev(sum(d$pos[d$sex == s]), sum(d$sex == s))))),
  do.call(rbind, lapply(sort(unique(d$age_generation)), function(g)
    data.frame(Group = "Age", Category = as.character(g),
               Result = fmt_prev(sum(d$pos[d$age_generation == g]),
                                 sum(d$age_generation == g)))))
)
print(b4, row.names = FALSE)
```

```
########## B4: Prevalence with Wilson 95% CI ##########
   Group Category                       Result
 Overall        - 186/1017 (18.3) [16.0-20.8]
     Sex        m   68/475 (14.3) [11.5-17.8]
     Sex        f  118/542 (21.8) [18.5-25.4]
     Age       20    19/145 (13.1) [8.6-19.6]
     Age       30    25/181 (13.8) [9.5-19.6]
     Age       40    20/160 (12.5) [8.2-18.5]
     Age       50    19/139 (13.7) [8.9-20.4]
     Age       60   45/180 (25.0) [19.2-31.8]
     Age       70   58/212 (27.4) [21.8-33.7]
```

In [28]:
```
##############################################################################
## 12. 投稿済み原稿の数値との照合
##############################################################################
cat("\n########## 照合: 投稿済み原稿 vs 本スクリプト ##########\n")
chk <- function(item, published, computed, ok) {
  cat(sprintf("%-42s 原稿: %-22s 再現: %-22s %s\n",
              item, published, computed, ifelse(ok, "一致", "*** 不一致 ***"))) }

chk("全体ANA陽性率", "18.3% (186/1017)",
    sprintf("%.1f%% (%d/%d)", 100*mean(d$pos), sum(d$pos), nrow(d)), TRUE)
chk("男性 / 女性", "14.3% / 21.8%",
    sprintf("%.1f%% / %.1f%%", 100*mean(d$pos[d$sex=="m"]),
            100*mean(d$pos[d$sex=="f"])), TRUE)
chk("Model 2 女性OR", "1.72 (1.23-2.40)",
    sprintf("%.2f (%.2f-%.2f)", exp(coef(m2)["sexff"]),
            exp(confint(m2)["sexff",1]), exp(confint(m2)["sexff",2])), TRUE)
chk("Model 3 女性OR", "1.87 (1.02-3.57)",
    sprintf("%.2f (%.2f-%.2f)", exp(coef(m3)["sexff"]),
            exp(confint(m3)["sexff",1]), exp(confint(m3)["sexff",2])), TRUE)
chk("Model 3 アレルギーOR", "1.13 (0.60-2.05)",
    sprintf("%.2f (%.2f-%.2f)", exp(coef(m3)["allergywith"]),
            exp(confint(m3)["allergywith",1]),
            exp(confint(m3)["allergywith",2])), TRUE)
chk("Table 2 喫煙/アレルギー見出し", "31 (6.6%)",
    sprintf("%d (%.1f%%)", sum(cc$pos), 100*mean(cc$pos)), FALSE)
chk("Figure 1 全体 P値", "P = 0.015 (6点回帰)",
    sprintf("quad P = %.3f", fit_all$P_quadratic[fit_all$Group=="Total"]), FALSE
chk("Figure 1 女性 P値", "P = 0.015 (6点回帰)",
    sprintf("quad P = %.3f", fit_all$P_quadratic[fit_all$Group=="Female"]), FALS
chk("Figure 1 男性 P値", "P = 0.121 (非有意)",
    sprintf("linear P = %.4f", fit_all$P_linear[fit_all$Group=="Male"]), FALSE)
cat("\n【不一致の解釈】\n",
```

```
    "・Table 2 の 31 は集計誤り。正しくは 56 (12.0%)。\n",
    "・Figure 1 は年齢群平均6点への当てはめだったため、個票解析とは結果が異なる。\n",
    "  男性は個票では有意（P=0.0008）。全体のU字は個票では消える（P=0.24）。\n")
```

```
########## 照合: 投稿済み原稿 vs 本スクリプト ##########
全体ANA陽性率                              原稿: 18.3% (186/1017)      再現: 18.3% (18
6/1017)       一致
男性 / 女性                                原稿: 14.3% / 21.8%          再現: 14.3% /
21.8%          一致
Waiting for profiling to be done...

Waiting for profiling to be done...

Model 2 女性OR                             原稿: 1.72 (1.23-2.40)       再現: 1.72
(1.24-2.40)       一致
Waiting for profiling to be done...

Waiting for profiling to be done...

Model 3 女性OR                             原稿: 1.87 (1.02-3.57)       再現: 1.86
(1.01-3.55)       一致
Waiting for profiling to be done...

Waiting for profiling to be done...

Model 3 アレルギーOR                       原稿: 1.13 (0.60-2.05)       再現: 1.14 (0.6
0-2.07)       一致
Table 2 喫煙/アレルギー見出し     原稿: 31 (6.6%)              再現: 56 (12.0%)
*** 不一致 ***
Figure 1 全体 P値                          原稿: P = 0.015 (6点回帰) 再現: quad P = 0.
241         *** 不一致 ***
Figure 1 女性 P値                          原稿: P = 0.015 (6点回帰) 再現: quad P = 0.
033         *** 不一致 ***
Figure 1 男性 P値                          原稿: P = 0.121 (非有意)  再現: linear P =
0.0008      *** 不一致 ***
Table 2 喫煙/アレルギー見出し     原稿: 31 (6.6%)              再現: 56 (12.0%)
*** 不一致 ***
Figure 1 全体 P値                          原稿: P = 0.015 (6点回帰) 再現: quad P = 0.
241         *** 不一致 ***
Figure 1 女性 P値                          原稿: P = 0.015 (6点回帰) 再現: quad P = 0.
033         *** 不一致 ***
Figure 1 男性 P値                          原稿: P = 0.121 (非有意)  再現: linear P =
0.0008      *** 不一致 ***
```

【不一致の解釈】
・Table 2 の 31 は集計誤り。正しくは 56 (12.0%)。
・Figure 1 は年齢群平均6点への当てはめだったため、個票解析とは結果が異なる。
　男性は個票では有意（P=0.0008）。全体のU字は個票では消える（P=0.24）。

In [29]:

```
###########################################################################
## 13. 出力
###########################################################################
wb <- createWorkbook()
addWorksheet(wb, "Table1");     writeData(wb, "Table1", t1)
addWorksheet(wb, "Table2");     writeData(wb, "Table2", t2)
addWorksheet(wb, "Table3");     writeData(wb, "Table3", t3)
addWorksheet(wb, "Table4");     writeData(wb, "Table4", t4)
addWorksheet(wb, "Fig1_model"); writeData(wb, "Fig1_model", fit_all)
addWorksheet(wb, "Fig1_curve"); writeData(wb, "Fig1_curve", pred_all)
```

```
addWorksheet(wb, "Fig1_obs");   writeData(wb, "Fig1_obs", obs_all)
addWorksheet(wb, "Prevalence_CI"); writeData(wb, "Prevalence_CI", b4)
saveWorkbook(wb, OUT_XLSX, overwrite = TRUE)

cat("\n\n=== 完了 ===\n")
cat("出力:", OUT_XLSX, "/", OUT_TIFF, "/", OUT_LOG, "\n")
sink()
```

```
=== 完了 ===
出力: Results_tables.xlsx / Figure1_revised.tiff / analysis_log.txt
出力: Results_tables.xlsx / Figure1_revised.tiff / analysis_log.txt
```

In [ ]:
